# Ourselves as Others See Us

**Published:** 1893-12

**Authors:** S. S. Towler

**Affiliations:** Marienville, Pa.


ARTICLE VII.

### OURSELVES AS OTHERS SEE US.

S. S. TOWLER, M. D., MARIENVILLE, PA.

Some one, I think it was Henry Ward Beecher, has said—"Many a man thinks he has religion when he only has dyspepsia, and many a man thinks he is going straight to the devil when he is only billious."

Some things have happened now and again in the experiences of the past, that lead me to suggest in this brief paper that, more or less frequently, the diagnosis is wrong because the practitioner has dyspepsia, and the prognosis gloomy because the doctor is bilious. How often have we seen the generally bright physician mistaken in the most unaccountable manner, and how often our own ideas have undergone a complete change, without a restudy of of the case or apparent cause. To-day your medical friend prescribes well and accurately, next day or next week, he mixes things up worse than a compounder of "patent cure-alls." To-day your patient or mine, is given scant courtesy and cold comfort, tomorrow, the same patient in the same condition is given attention and a hopefulness that does him a 'world of good." And you and I thought on each of the separate days that we were doing the correct thing. How much was *our* stomach and *our* liver to blame for the difference in the patient's condition from our own point of view. Is a practitioner, whose stomach is rebelling against his own foolishness, in a fit condition to judge nicely of the infirmities of his patient—Is the doctor with a torpid liver capable of making a cheerful prognosis? As a rule I don't believe he is, and I have no doubt that our own physical condition has much to do with the success of our treatment, though the ailment—personally—may be so slight as to pass unnoticed, except by the thought of, "I don't feel well."

I don't propose slopping over in a long-winded article 'analyzing' this condition, I simply want to draw attention to it "for the good of the Order," on the principle that we learn by "line upon line, precept upon precept, here a little and there a little, etc."

The great and good surgeons and gynecologists of the day, insist that personal cleanliness of the surgeon himself is of vital importance to successful operation. Let us carry the idea still further and learn that personal healthfulness—a clean stomach and an active liver—are of vital importance in the line of general therapeutics. Let us go a step further and learn for ourselves, that if we would be the educators in hygiene, etc., that we claim to be, then example must follow precept. This means that, indigestible trash, no matter how toothsome," must be banished from our table, side-board, banquet and reception, and with it should go by all means "old Frumenti," and all his kindred spirits. It means that, *our* back yard,

*our* alley, *our* vaults, *our*selves and *our* belongings must be kept consistent with our teaching and advice to others.

There is nothing new in this (more's the pity) that so little attention is paid to knowledge acquired. Hundreds of physicians have read the editorial, "The doctor and the cook." If ten per cent. of them put it in practice, there will be more stars in professional crowns than the editor ever fondly dreamed of. Said an elder in the church, "had it not been for the inconsistencies of christians themselves, Christianity would have swept the world long ago." So with "ourselves," had it not been for personal and persistent violation of our own code, we would have doubled our power for the physical good of our generation.

What student fresh from fields and woods, doomed to six hours of lectures, *per diem*, in a poorly-ventilated room, goes out of it with a clear idea of two per cent. of what has been uttered, and in my student days, woe to him if the Professor caught him napping. A recollection comes to me now, of a wag punching his sleepy neighbor in the lecture room, with his elbow and the remark, "Wake up, Jim, you have just missed one of the old man's real nasty stories."

What practitioner cannot recall, (if he is honest with himself) the fact, that, his own supper was more to blame for the weary hours and his own crankiness beside the slow obstetric, than any "foolishness of the patient. How many doctors blame the out-house vault for the fatal ending of the case, when their own is in as bad condition. How many "scold" the patient for undue exposure, when they, themselves, have not thought enough or pride enough, to mend the soles of their own shoes. How many talk about—"no wonder she died with so much dirt in the house"—when their own office is akin to a poorly kept pigpen. What importance can a doctor expect to be given by a patient to his directions as to "diet," while he is a persistent violator of his own rules, or what lasting impression can a professor make on his students when he, and all the rest of us in the Fraternity with him, show contempt for the advice given, at the next Society banquet.

We all have been greatly concerned that the coming medical man should be more competent than he of the present and, what with the new Examiner's Bill and improved methods of teaching in the colleges, the State Society may be fairly said to have done its duy by the practitioner of the future.

Now suppose we take a survey of ourselves and see if we cannot be more of a power for good, and advance the dignity of a great profession by personal and collective consistency.

Every thinking man knows that, no course of lectures, no matter how ably delivered; no clinics, no matter how expert the demonstration; no bed-side exposition, no matter how clearly brought out, can give a man good judgment or sound common sense. BUT there is one thing we *can all do*, and that is this—observe our own dietetic and hygienic laws personally, and give what brains we have a chance.

Let us be *practitioners indeed.—Medical and Surgical Reporter*.

---

ARTICLE VIII.

